# Effect of *Ascaris Lumbricoides* specific IgE on tuberculin skin test responses in children in a high-burden setting: a cross-sectional community-based study

**DOI:** 10.1186/1471-2334-12-211

**Published:** 2012-09-11

**Authors:** Nelda van Soelen, Anna M Mandalakas, H Lester Kirchner, Gerhard Walzl, Harleen M S Grewal, Marc Jacobsen, Anneke C Hesseling

**Affiliations:** 1 Desmond Tutu TB Centre, Department of Paediatrics and Child Health, Faculty of Health Sciences, Stellenbosch University, Tygerberg, South Africa; 2 Section on Retrovirology and Global Health, Department of Pediatrics, Baylor College of Medicine, Houston, TX, USA; 3 Center for Global Health, Texas Children’s Hospital, Houston, TX, USA; 4 Division of Medicine, Geisinger Clinic, Danville, PA, USA; 5 Immunology Research Group, Department of Biomedical Sciences, Stellenbosch University, Tygerberg, South Africa; 6 Section of Microbiology and Immunology, The Gade Institute, University of Bergen and Department of Microbiology, Haukeland University Hospital, Bergen, Norway; 7 Deptartment of General Pediatrics and Neonatology, University Childrens Hospital, Duesseldorf, 40225, Germany

**Keywords:** Tuberculosis, Helminth infection, *Ascaris, M.tb* infection, Immune polarization, Paediatric tuberculosis

## Abstract

**Background:**

*M.tuberculosis* (*M.tb*) is associated with enhanced T helper cell type 1 (Th1) immune responses while helminth infection is associated with T helper cell type 2 (Th2) immune responses. Our aim was to investigate whether helminth infection could influence the ability to generate an appropriate Th1 immune response that is characterized by a positive tuberculin skin test (TST), in *M.tb* exposed children.

**Methods:**

We completed a community-based, cross sectional household contact tracing study, using matched enrolment of HIV negative children with and without documented household *M.tb* exposure. We documented demographics, clinical characteristics, HIV status, *M.tb* exposure (using a standard contact score) and *M.tb* infection status (TST > = 10 mm). *Ascaris lumbricoides-*specific IgE was used as proxy for *Ascaris* infection/exposure.

**Results:**

Of 271 children (median age 4 years (range: 4 months to 15 years)) enrolled, 65 participants (24%) were serum positive for *Ascaris* IgE. There were 168 (62%) children with a documented household tuberculosis contact and 107 (40%) were (TST) positive overall.

A positive TST was associated with increasing age (Odds Ratio (OR) =1.17, p < 0.001), increasing *M.tb* contact score (OR = 1.17, p < 0.001), previous tuberculosis treatment (OR = 4.8, p = 0.06) and previous isoniazid preventive treatment (OR = 3.16, p = 0.01). A visible bacillus Calmette-Guérin (BCG) scar was associated with reduced odds of being TST positive (OR = 0.42, p = 0.01).

*Ascaris* IgE was not associated with TST status in univariate analysis (OR = 0.9, p = 0.6), but multivariable logistic regression analysis suggested an inverse association between *Ascaris* IgE status and a positive TST (OR = 0.6, p = 0.08), when adjusted for age, and *M.tb* contact score. The addition of an age interaction term to the model suggested that the age effect was stronger among *Ascaris* IgE positive children; the effect of being *Ascaris* IgE positive significantly reduced the odds of being TST positive amongst younger children while this effect weakened with increasing age.

**Conclusions:**

Our preliminary findings highlight a high prevalence of both *Ascaris* exposure/infection and *M.tb* infection in children in an urban setting. Helminth exposure/infection may reduce the immune response following *M.tb* exposure when controlling for epidemiological and clinical covariates. These findings might be relevant to the interpretation of immunological tests of *M.tb* infection in children.

## Background

Infectious diseases are important causes of morbidity and mortality in children living in the developing world. Tuberculosis is a major global threat with an estimated 9.4 million incident cases reported globally in 2009 [1], with South Africa ranked third with an estimated annual notification rate of 971 per 100 000 per year [1]. Paediatric tuberculosis contributes an estimated 15-20% of the overall global caseload, with >80% cases notified from the 22 so-called “high-burden” countries, including South Africa [2]. Young children exposed to and infected with *Mycobacterium tuberculosis* (*M.tb*) are at high risk for disease progression in the absence of effective chemoprophylaxis [3].

Poor socio-economic circumstances, prevalent in many settings with a high burden of tuberculosis, also predispose to poor living conditions, overcrowding and increased risk of helminth exposure and infection [4,5]. Helminth infection in children is often chronic and is associated with anaemia, growth retardation, impaired cognitive functioning and chronic inflammatory diseases [6-8]. The World Health Organization (WHO) classifies South Africa as one of the 130 helminth-endemic countries (prevalence of infection > 20% - 50%) [9]. The WHO Integrated Management of Childhood Illness (IMCI) guidelines therefore routinely recommend regular deworming in children older than 1 year [10]; however, this is not consistently practiced and the constant risk of re-infection remains high in helminth-prevalent settings [4,5].

*M.tb* exposure involves complex immune processes and leads to a spectrum of infection and clinical disease outcomes in children, including *M.tb*-exposed but uninfected, infected non-diseased and diseased [11,12]. In addition to innate host immunity, protection against *M.tb* requires an effective adaptive cellular immune response characterized by robust T helper cell type 1 (Th1) T-cell immunity and relative weaker T helper cell type 2 (Th2) T-cell immune responses [13-17]. The tuberculin skin test (TST), the most established clinical-epidemiological immune measure of *M.tb* infection, is a marker of a Th1-type delayed type hypersensitivity reaction. Previously *M.tb*-sensitized circulating lymphocytes are attracted to the intra-dermal tuberculin injection site, followed by monocyte, macrophage, CD4 + − and CD8+ T-lymphocyte accumulation and release of inflammatory mediators that causes clinical erythema and oedema [12].

Th2 cells produce cytokines which play a role in protection against extracellular organisms and are increased in the presence of helminth infection [18-21], specifically *Ascaris lumbricoides *[20] and hookworm species [20,21]. Data regarding the role of Th2 cells in the presence of *Trichiuris trichiura* infection are inconsistent [15,22]. Th2 cell types impair signal transduction and induce anergy of immune cells, which might decrease the ability to generate protective cellular immunity to protect against common infections, like *M.tb* and to routine vaccinations [19]. Helminth infection is further associated with suppressive T-cell population induction and inhibitory cytokine production which can suppress Th1-type responses and interfere with effector T-cell activation, potentially resulting in altered memory immune responses against *M.tb *[15].

The current limited evidence supports a potential immune modulating effect of helminths on mycobacterial immune responses in humans [15,21,23], although contradictory evidence also exists [24,25]. We therefore investigated the effect of an *Ascaris-*specific IgE positive status on a commonly used marker of *M.tb* infection (TST), in children in a setting with a high burden of tuberculosis. We hypothesized primarily that helminth infection in children, while controlling for *M.tb* exposure and other relevant covariates, would decrease the ability to generate an appropriate Th1 immune response characterized by a positive TST in children.

## Methods

This was a prospective cross-sectional community-based study. Study participants were recruited from three high burden tuberculosis communities: Ravensmead, Uitsig (R/U) and Site C, Khayelitsha (Site C), in Cape Town, Western Cape Province, South Africa. The communities are demographically heterogeneous, and respectively represent ethnic groups that are predominantly of South African Mixed (R/U) and Xhosa African ancestry (Site C).

The tuberculosis notification rate in the province was 994 per 100 000 in adults and 671 per 100 000 in children aged 0 – 14 years in 2008 (Western Cape Department of Health, unpublished data). There is near universal infant bacille Calmette-Guérin (BCG) vaccination.

Prior surveys from school-going children in R/U found that 25% were stool positive for *Ascaris lumbricoides* and 51% for *Trichuris trichiura *[26]. Routine clinic and school-based deworming has since become standard of care in local health care centres. There are currently no published data on the prevalence of helminth infection in children in Khayelitsha.

Participants were recruited following identification of consenting adults (≥ 18 years of age), who routinely started tuberculosis treatment in the preceding 3 months (“source case”) at the local clinic. Participants were eligible for enrolment if they were a household child contact between 3 months and 15 years of age. Households were identified through TB source cases (“tuberculosis households”), and matched neighbouring households without known tuberculosis exposure (“neighbouring households”). The ratio between tuberculosis and neighbouring households was approximately 1:1.

We identified tuberculosis households within 2–4 weeks following the adult source case identification, then completed a home visit and collected data on *M.tb* exposure and demographic characteristics. In addition, all adult household members were screened for symptoms of tuberculosis followed by sputum collection, if they were symptomatic.

Following written informed consent from the parents/caregivers, children aged 3 months – 15 years who were not currently on tuberculosis therapy, were enrolled in the study. Children were deferred for enrolment if there was a) prior TST administration in the preceding 3 months, b) live measles or polio vaccine within the past 6 weeks or c) recent acute illness requiring hospitalization (preceding 6 weeks). Children on isoniazid preventive therapy (IPT) were not excluded, whereas HIV-infected children were.

All children were investigated for *M.tb* infection and disease using standard protocols [27,28], including TST, a standard symptom questionnaire , blinded independent dual expert review of chest radiographs (postero-anterior and lateral), sputum or gastric washing for mycobacterial culture (Mycobacterial Growth Indicator Tubes; Becton Dickinson, Sparks, USA), a TST, and clinical examination.

The TST (Mantoux method; 2 tuberculin units purified protein derivative; Statens Serum Institute, Copenhagen) was administered intra-dermally on the volar aspect of the left forearm by trained standardized study nurses, and read within 48–72 hours using the ball-point and ruler method and callipers. The TST was regarded as positive if measuring ≥10 mm in HIV-negative children for clinical care. Unless already known to be HIV-infected, participants were tested for HIV (Abbott Determine Rapid HIV test), followed by a confirmatory laboratory based ELISA/PCR test if positive or indeterminate.

Data were collected to quantify the degree of *M.tb* exposure (*M.tb* contact score) [29]. In the absence of a gold standard of infection, we used this score to quantify the degree of exposure in children as a surrogate reference standard for *M.tb* infection [29-31] [Table 1. The score includes measures of source case infectivity, the average reported duration of exposure to the source case, and the child’s reported proximity to the source case. TST results in children from the study communities were previously shown to be well correlated with this score [29,30].

**Table 1:** **Paediatric Mycobacterium tuberculosis contact score incorporating measures of infectivity, proximity and infectivity of documented exposure **[[29]]

	
1	Is the index case the child’s mother?
2	Is the index case the child’s primary caretaker?
3	Does the index case sleep in the same bed as the child?
4	Does the index case sleep in the same room as the child?
5	Does the index case live in the same household as the child?
6	Does the index case see the child every day?
7	Is the index case coughing?
8	Does the index case have reported pulmonary tuberculosis?
9	Does the index case have smear positive sputum?
10	Is there more than one index case in the child’s house hold?

Note: a score of zero is assigned in the absence of any documented M.tb exposure.

We also recorded information on BCG vaccination history and scar presence, previous and current isoniazid preventive therapy, previous tuberculosis treatment, nutritional status (z-scores), household composition, measures of socio-economic status (household assets), and reported history of deworming during the preceding 6 months. Z-scores were calculated with reference to the 2006 World Health Organization Child Growth Standards for children up to five years of age and the 2000 Centres for Disease Control and Prevention growth charts reference population for children that were older than 5 years [32]. Reported socio-economic indicators were based on surveys in our study communities and were used in the absence of reliable data on reported income, given high unemployment rates in these impoverished urban communities.

Sensitization to *Ascaris lumbricoides* was determined by the ImmunoCAP specific-IgE testing which measures circulating *Ascaris-*specific IgE concentration in serum. This method was developed for clinical diagnosis and laboratory use where reagins belonging to the IgE antibody class, which develop shortly after exposure, are measured by a fluoroenzymeimmunoassay [33]. Results are expressed as kU/L and are typically categorised in 6 groups (Table 2) or interpreted as positive or negative based on a binary classification (25). 

**Table 2:** **Distribution of *Ascaris*-specific IgE values by RAST classification in children (n = 271)**

**RAST rating**	**IgE level (kU/L)**	**Frequency**	**Row percentage**
0	< 0.35^1^	206	76.01
1	0.35 - 0.69^2^	17	6.30
2	0.70 - 3.49^3^	31	11.40
3 +	3.50 + ^4, 5, 6, 7^	17	6.27
All	*Ascaris*-specific IgE positive^8^	65	24.00

^1^Absent or undetectable allergen specific IgE.

^2^Low level of allergen specific IgE.

^3^Moderate level of allergen specific IgE.

^4^High level of allergen specific IgE: 3.50 - 17.49.

^5^Very high level of allergen specific IgE: 17.50 - 49.99.

^6^Very high level of allergen specific IgE: 50.0 - 100.00.

^7^Extremely high level of allergen specific IgE: > 100.00.

^8^ Any RAST response > 0.34 kU/L.

### Data analysis

Data analysis was performed using STATA/SE version 12.0 (StataCorp LP, StataCorp, 4905 Lakeway Drive, College Station, Texas 77845 USA). An alpha level of less than or equal to 0.05 was regarded as statistically significant. The main outcome of interest was *M.tb* infection, measured by a positive TST (≥10 mm). The main exposure of interest, *Ascaris* IgE, was categorised according to RAST categories and dichotomized (positive if ≥0.35kU/L). This measure, consistent with previous work (25) was used as a measure of *Ascaris* infection or sensitization.

We assessed the effect of predictors of *M.tb* infection in univariate analysis, with the primary determinant being *Ascaris* IgE status in 271 enrolled participants. Child baseline characteristics were described overall, and stratified according to *Ascaris* IgE status for baseline characteristics. Continuous data were described using mean and standard deviations and median and inter-quartile ranges, dependent on data distribution. Categorical variables were described using frequencies and percentages.

We developed logistic regression models that included *Ascaris* IgE status, age and the *M.tb* contact score. These measures were significant during univariate analysis or were clinically or epidemiologically relevant as covariates. Additionally, the developed model was examined for interactions between the explanatory and environmental variables in order to establish if possible effect modification is present. Results of the regression model are presented as adjusted odds ratios (aORs) and corresponding 95 percent confidence intervals (95% CIs).

We complied with the Helsinki Declaration and the study was approved by the Health Research Ethics Committee of the Stellenbosch University (reference number N05/07/129 and N08/08/207).

## Results

Of 337 total child participants enrolled, 271 HIV-negative children were included in the analysis (n = 66, 20% were HIV infected and excluded). The median age was 4 years (range: 4 months to 15 years).

Few children (2%) were receiving IPT at the time of enrolment despite the fact that 67% of children were household contacts of a tuberculosis index case currently on therapy. Of children with a documented contact (n = 181), 121 were below 5 years of age and were referred for IPT. Previous IPT use was reported in 9% and 3% had previously received tuberculosis treatment.

Housing and socio-economic indicators indicate the poor living conditions prevalent in these communities: almost 20% of participants did not have access to a fridge or radio at home while almost 5% relied occasionally on selling household assets. Only 68% of children lived in a house with a flushing toilet and 83% had access to piped water in the household. The majority of children resided in formal brick housing (77%) [Table 3].

**Table 3:** Characteristics of child participants (n = 271)

**Participant characteristics**	**All participants (n = 271)**	***Ascaris*IgE negative (n = 206)**	***Ascaris*IgE positive (n = 65)**	**TST negative (<10 mm, n = 64)**	**TST positive (≥10 mm, n = 107)**
Age (median, inter-quartile range(IQR))	4.4 (1.9 – 8.3)* §§	3.5 (1.6 – 6.7)	6.6 (4.5 - 10.3)	3.5 (1.6 – 5.7)	6.1 (3.2 – 10.2)
*M.tb* contact score (median (IQR))	4 (0 – 5)* §§	4 (1 – 6)	3 (0 – 5)	4 (0 – 5)	5 (1 – 7)
Male gender (%)	111 (41.0)	82 (39.8)	29 (44.6)	68 (41.5)	43 (40.2)
Ethnicity (%)					
African	55 (20.3)	47 (22.8)	8 (12.3)	32 (19.5)	23(21.5)
Mixed ancestry	216 (79.7)	158 (76.7)	57 (87.7)	132 (80.5)	84 (78.5)
BCG history or scar present (%)	221 (81.6) §§	172 (83.5)	49 (75.4)	143 (87.2)	78 (74.3)
Previous TB treatment (%)	8 (3.0) §§	5 (2.4)	3 (4.6)	2 (1.2)	6 (5.6)
Current IPT (%)	4 (1.5)	3 (1.9)	0	2 (1.2)	2 (1.9)
TST positive (%)	107(39.5)	83 (40.3)	24(36.9)	NA	NA
History of passing a worm in <3 months (%)	21 (7.8)	13 (6.3)	7 (87.5)	15 (9.2)	6 (5.6)
History of deworming in < 3 months^3^ (%)	18 (6.6)	11 (5.3)	8 (12.3)	13 (7.9)	5 (4.7)
Anthropometric data (mean (SD))					
Weight for age	−0.55 (1.20)	−0.49 (1.2)	−0.76 (1.3)	−0.48 (1.3)	−0.67 (1.1)
Height for age	−1.29 (1.6)	−1.27 (1.6)	−1.34 (1.5)	−1.20 (1.9)	−1.42 (0.9)
Weight for height	0.35 (1.2)	0.40 (1.2)	0.13 (1.1)	0.41 (1.2)	0.22 (1.1)
**Socio-economic indicators and household demographics**					
Presence of a radio in household (%)	217 (80.1) §§	163 (79.)	54 (83.1)	139 (84.8)	78 (72.9)
Presence of a refrigerator in household (%)	221 (81.6) §§	166 (80.6)	55 (84.6)	144 (87.8)	77 (72.0)
Relying on the sale of household assets (%)	13 (4.8)	12 (5.8)	1 (1.5)	8 (4.9)	5 (4.7)
Flush toilet in the house (%)	184 (67.9)	141 (68.5)	43 (66.2)	114 (69.5)	70 (65.4)
Piped running water in residence (%)	225 (83.0)	169 (82.0)	56 (86.2)	137 (83.5)	88 (82.2)
Housing structure (%)					
Tin shack	36 (13.4)	30 (14.6)	6 (9.5)	19 (1.7)	17 (16.1)
Brick	205 (76.5)	154 (75.1)	51 (81.0)	128 (78.5)	77 (73.3)
Other	27 (10.1)	21 (10.2)	6 (9.5)	16 (9.8)	11 (10.5)
Household composition (median (IQR))					
Number of adults	5 (3 – 6)	5 (3 – 6)	4 (3 – 7)	5 (3 – 6)	4 (2 – 6)
Number of children	3 (2 – 5)	3 (2 – 5)	4 (2 – 5)	4 (2 – 5)	3 (2 – 4)
Median ratio of adult:children	1.25 (1 – 1.8)	1.25 (1 – 1.8)	1.25 (1–1.8)	1.25 (1 – 1.9)	1.25 (1 – 1.8)

TB = tuberculosis; BCG = bacillus Calmette-Guérin; IPT = Isoniazid preventive therapy, TST = tuberculin skin test.

*Ascaris groups significantly different, P-value ≤0.05.

§§ TST groups significantly different, P-value ≤0.05.

*Ascaris* IgE results are described in Table 2. A total of 65 (24%) children were *Ascaris* IgE positive. Of these, 37% were also TST positive. Most Ascaris IgE positive participants were within RAST range 2 (moderate level of allergen specific IgE) followed by ranges 1 (low level of allergen specific IgE) and 3 (high level of allergen specific IgE) [Table 2]. Of the 8% of children in whom the caregiver reported that the child had passed a worm in the preceding 3 months, most (86%) had been dewormed. Anthropometric measures were not associated with either positive Ascaris or TST status.

Overall, 40% of children were TST positive at enrolment. Children were more likely to have a positive TST if they were older (OR 1.2, p < 0.001), had a higher *M.tb* contact score (OR 1.2, p < 0.001), had received previous TB treatment (OR 4.8, p = 0.06) or previous IPT (OR 3.2, p = 0.01). A visible BCG scar (OR 0.4, p = 0.01) was associated with reduced odds of infection [Table 4]. There was no significant association between positive *Ascaris* IgE and positive TST status (OR = 0.9, 95% CI 0.5– 1.6, p = 0.63).

**Table 4:** **Univariate analysis of predictors for *Ascaris*-specific IgE positivity (n = 271)**

	***Ascaris *IgE positive (n = 65)**		
	**OR**	**95% CI**	**p-value**
Age ^1^	1.20	1.11 – 1.29	<0.001
TB contact score^1^	0.88	0.80 – 0.98	0.02
Male gender	1.22	0.69 – 1.14	0.49
Ethnicity African	0.47	0.21 – 1.06	0.07
Mixed ancestry	2.12	0.94 – 4.76	0.07
BCG history or scar present	0.63	0.32 – 1.25	0.18
Previous TB treatment	1.95	0.45 – 8.37	0.37
History of recent worm	2.08	0.82 – 5.28	0.12
History of recent deworming (< 3 months)	1.27	0.10 – 16.81	0.86
Z-scores Weight for age	0.81	0.63 – 1.05	0.11
Height for age	0.97	0.80 – 1.17	0.76
Weight for height	0.81	0.60 – 1.10	0.18
**Socio-economic indicators and household demographics**			
Presence of a radio in the household	1.30	0.62 – 2.69	0.49
Presence of a refrigerator in the household	1.33	0.62 – 2.83	0.47
Relying on the sale of household assets	0.25	0.03 – 1.98	0.19
Access to flush toilet facility in the house	0.90	0.50 – 1.63	0.73
Piped running water in the house	1.36	0.62 – 3.00	0.44
Living in a brick house	1.23	0.63 – 2.40	0.54
Household composition			
Number of adults in household	0.01	0.90 – 1.14	0.83
Number of children in household	1.08	0.94 – 1.25	0.28
Median ratio of adults:children	0.98	0.74 – 1.31	0.91

BCG, bacillus Calmette-Guérin; TB, tuberculosis.

Based on the univariate analysis, we included age, *M.tb* contact score and binary *Ascaris* IgE status in a regression model that demonstrated an increased risk for a positive TST in older children (aOR 1.2, 95% CI 1.1 – 1.3) and those with a higher *M.tb* contact score (aOR 1.2, 95% CI 1.1 – 1.3). This model also showed an inverse association between TST and *Ascaris* IgE positivity (aOR 0.6, 95% CI 0.1 – 1.1).

A second model including *M.tb* contact score, age, *Ascaris* IgE and an age interaction term showed that the effect of age was stronger among those children with a positive *Ascaris* IgE status. Being *Ascaris* IgE positive significantly reduced the odds of being TST positive amongst younger children, while this effect weakened as age increased (Table 5).

**Table 5:** Multivariable logistic regression model of predictors of TST positivity in children

**Model 1:**		
	**aOR (95% CI)**	
Age	1.22 (1.13 – 1.32), p < 0.001	
TB contact score	1.21 (1.09 – 1.34), p < 0.001	
*Ascaris* IgE positive	0.55 (0.28 – 1.07), p = 0.08	
**Model 2: Interaction between Helminth Positive/Negative and Age**		
TB contact Score		1.22 (1.10 - 1.34)
Age effect		
	IgE negative	1.17 (1.08 - 1.27)
	IgE positive	1.44 (1.21 - 1.73)
IgE effect		
	25% age	0.18 (0.05 - 0.67)
	50% age	0.30 (0.11 - 0.78)
	75% age	0.67 (0.32 - 1.39)

aOR = adjusted Odds ratio, 95% CI = 95 percent confidence interval.

## Discussion

We demonstrate a high prevalence of *M.tb* and *Ascaris lumbricoides* infection in children from households with and without documented tuberculosis exposure living in poor urban South African communities. Our results suggest an inverse relationship between *Ascaris* IgE and TST positive status, which is consistent with our hypothesis.

False-negative TST results might be due to technical factors or a suppressed immune status while false-positive tests may be attributed to BCG vaccination and the effect of non-tuberculous mycobacteria [12]. In our study, a positive TST was associated with increasing age and an increasing *M.tb* contact score, which is biologically plausible and consistent with other data [30,34]. The presence of a BCG vaccination scar [35-37] and indicators of higher socio-economic household status [38] protected against having a positive *M.tb* infection phenotype.

Our multivariable logistic regression adjusting for age, degree of *M.tb* exposure and previous TB therapy, supports this hypothesis; children with a positive *Ascaris* IgE appeared to be less likely to mount a positive TST response, especially if they were younger.

In the absence of stool samples, as in this study, serum *Ascaris*-induced specific IgE is widely used as proxy for *Ascaris* infection. *Ascaris* IgE however cannot differentiate previous infection from new infection and/or previous sensitization, and therefore assumes *Ascaris* infection and Th2 polarization without information regarding the time of infection [39], similar to measures of *M.tb* infection. Additionally potential cross-reactivity due to *Trichiuris trichiura* infection cannot be excluded [40]. Despite these limitations, *Ascaris* IgE responses remain a useful proxy for immunological and epidemiological studies, and are a standard and widely used indication of *Ascaris* exposure/infections.

Despite universal clinic access to deworming and school- based deworming programs, the positive *Ascaris* IgE levels of 24%, although lower than previously reported from these communities [26], are high and consistent with WHO definitions for a helminth endemic country [9]. Deworming may not be practised as regularly as required (at least once a year), as indicated by the low proportion of caregivers (7%) who had reported deworming of the child in the preceding 3 months. On the other hand, helminth re-exposure and infection, given the poor socio-economic circumstances is also possible [9,38]. These factors should be investigated in longitudinal studies with repeat measures of infection.

Older children (OR 1.2, p < 0.001) were more likely to be *Ascaris* positive which we attribute to the age-related risk of infection and immune system responsiveness, and possibly due to behavioural patterns (i.e. more outdoor activities in shared environments like schools and sports fields).

Few children were on IPT at enrolment which could reflect the active contact tracing strategy used by the study; however previous studies from these communities indicate poor IPT uptake and adherence in young children under routine programmatic conditions [41-43].

This study is limited by its cross-sectional nature, the limited sample size and the exclusion of HIV-infected children, due to small numbers. The enrolment strategy was purposeful to ensure a high proportion of children with recent *M.tb* exposure and included children from neighbouring “control” households as a measure of background *M.tb* transmission. Finally, the proxy used for *Ascaris* in our study does not apply to all potential helminth species, and might underestimate the general and differential effect of helminth infection. Deworming was only reported on a questionnaire basis and its impact on mycobacterial and other immune responses could therefore not be rigorously evaluated.

In light of these limitations, these results need to be verified in paediatric cohort studies, preferably with additional measures of helminth infection including stool samples and the standard evaluation of the effect of deworming on mycobacterial immune responses.

## Conclusions

The results support our hypothesis that helminth infection may change immune responses in children. This may play a clinically measureable role in widely used immune diagnostic tests that measures *M.tb* infection, especially in high prevalence tuberculosis and helminth infection settings.

## Abbreviations

95% CI: 95 percent confidence interval; aOR: Adjusted odds ratio; BCG: Bacillus Calmette-Guérin; IMCI: Integrated Management of Childhood Illness; IPT: Isoniazid preventive therapy; *M.tb:Mycobacterium tuberculosis*; OR Odds ratio; R/U: Ravensmead and Uitsig; Site C: Site C, Khayelitsha; TB: Tuberculosis; Th1: T helper cell type 1; Th2: T helper cell type 2; TST: Tuberculin skin test; WHO: World Health Organisation.

## Competing interests

All authors declare that they have no competing interests.

## Authors’ contributions

NVS was involved in study design, data analysis, interpretation and manuscript preparation and primarily responsible for manuscript writing. AMM participated in design, implementation, analysis and writing. HLK participated in analysis, interpretation and writing. GW participated in design, writing and interpretation. HMSG was involved in study design and interpretation. MJ participated in writing and interpretation. ACH participated as academic supervisor in all phases of the study and manuscript preparation including design, implementation, analysis and writing. All authors read and approved the final manuscript.

## Pre-publication history

The pre-publication history for this paper can be accessed here:

http://www.biomedcentral.com/1471-2334/12/211/prepub
